# Impact of vaccine hesitancy on secondary COVID-19 outbreaks in the US: an age-structured SIR model

**DOI:** 10.1186/s12879-022-07486-0

**Published:** 2022-06-01

**Authors:** Alfonso de Miguel-Arribas, Alberto Aleta, Yamir Moreno

**Affiliations:** 1 Institute for Biocomputation and Physics of Complex Systems (BIFI), University of Zaragoza, 50018 Zaragoza, Spain; 2 Department of Theoretical Physics, University of Zaragoza, 50009 Zaragoza, Spain; 3 ISI Foundation, Via Chisola 5, 10126 Turin, Italy

**Keywords:** COVID-19, Mathematical modeling, Age-structured SIR, Vaccination, Hesitancy

## Abstract

**Background:**

The COVID-19 outbreak has become the worst pandemic in at least a century. To fight this disease, a global effort led to the development of several vaccines at an unprecedented rate. There have been, however, several logistic issues with its deployment, from their production and transport, to the hesitancy of the population to be vaccinated. For different reasons, an important amount of individuals is reluctant to get the vaccine, something that hinders our ability to control and—eventually—eradicate the disease.

**Materials and methods:**

Our aim is to explore the impact of vaccine hesitancy when highly transmissible SARS-CoV-2 variants of concern spread through a partially vaccinated population. To do so, we use age-stratified data from surveys on vaccination acceptance, together with age-contact matrices to inform an age-structured SIR model set in the US.

**Results:**

Our results show that per every one percent decrease in vaccine hesitancy up to 45 deaths per million inhabitants could be averted. A closer inspection of the stratified infection rates also reveals the important role played by the youngest groups. The model captures the general trends of the Delta wave spreading in the US (July-October 2021) with a correlation coefficient of $\rho =0.79$.

**Conclusions:**

Our results shed light on the role that hesitancy plays on COVID-19 mortality and highlight the importance of increasing vaccine uptake in the population, specially among the eldest age groups.

## Background

After more than two years since the onset of the COVID-19 outbreak, firstly reported by the Chinese authorities on December 31, 2019, it is clear that this pandemic has become the worst one in at least a century.

Multiple aspects of our life have been severely affected at various scales: psychological [1, 2] and social [3]; human-related systems and infra-structures [4, 5]; supply chains [6, 7]; and the economy in general [8, 9]. To manage the disease, it was mandatory to adopt a plethora of measures aimed at reducing the mixing and interaction among individuals in order to mitigate SARS-CoV-2 transmission and propagation. Lockdowns [10–14], curfews and mobility restrictions [15–18], social distancing [19–21], personal protection [22, 23] are now part of the new normalcy across the world. This “new normal” [24–26], being its impact as critical as the virus itself, was conceived and promised as something that should be ephemeral, a toll to pay, just until the ultimate solution arrives: the vaccines.

A rapid and massive scientific effort [27, 28] to develop a vaccine against SARS-CoV-2 was deployed and successfully achieved in less than a year; another unprecedented fact. From December 2020, just a year after the onset of the pandemic, several nations started their vaccination campaigns in the pursue of herd immunity to control the pandemic. But again, further problems proliferated. From lack of confidence due to the relative short time for vaccine development and approval, to the fear of suffering serious side effects, or due to outlandish conspiracy theories, some people hesitate or are reluctant to vaccination [29–32]. For good or bad, this phenomenon is neither exclusive nor new [29, 33]. To be vaccinated (or not) is, in most countries, a choice of the individual, even though the consequences of such a choice go beyond the self and affect the social sphere. Hesitancy poses an ethical problem since if a critical fraction of individuals declines vaccine uptake for any disease, resurgence is to be expected [34–36]. This already happened in the UK, which was declared measles-free in 2017 but lost this status just 2 years later due to sub-optimal vaccination uptake [37].

Our aim is to quantify the effects of vaccine hesitancy in the US during the COVID-19 pandemic. In particular, given the large heterogeneity found across the US population, we perform our analysis on each state. It is worth noting that we do not intend to replicate the real trajectory of the COVID-19 pandemic in the US, neither we aim to accurately forecast the unfolding of future outbreaks and epidemic sizes. Instead, we focus on looking at correlations between variables related to hesitancy and the disease impact on the population, i.e., attack rates and deaths. We also explore the role that age structure plays in conditioning the outcomes and estimate potentially averted deaths if a 1% point more of the hesitant fraction of the population would change its attitude. Finally, we compare the model output from our hypothetical scenario with the epidemic impact caused during the COVID-19 Delta wave during July-November 2021.

## Materials and methods

We make use of an age-structured SIR model to simulate the spreading dynamics, which is fed with real and up-to-date data of the US age-distributed population and contact matrices, as well as with survey-based seroprevalence estimations [38]. We propose a hypothetical scenario in which COVID-19 outbreaks emerge in each state, independently, with a mitigated propagation due to the presence of some restrictions, while there is an ongoing vaccination campaign designed following the information obtained from public surveys [39]. Once the vaccination and this first outbreak end, we assume a *back to normal* situation, where all restrictions are lifted, disease awareness vanishes, and a new outbreak sets in. We assume that these successive outbreaks happen for a more transmissible variant of the virus, mimicking in this way the observed evolutionary path of the SARS-CoV-2 variants of concern.

### Epidemic model

Given the utmost relevance of age in the effects of COVID-19, it is compulsory to introduce the age distribution of the population and the specific interaction between age groups to adequately model the dynamics of the disease [40–42]. We use the estimated age-contact matrices provided by Mistry et al. [43] updated to the population structure of 2019 [44, 45]. Then, we build an age-structured SIR model defined by this set of equations [43]:

1 $$\begin{aligned} \frac{{dS_{a} }}{{dt}} & = - \lambda _{a} S_{a} , \\ \frac{{dI_{a} }}{{dt}} & = \lambda _{a} S_{a} - \gamma I_{a} , \\ \frac{{dR_{a} }}{{dt}} & = \gamma I_{a} , \\ \end{aligned}$$

where $S_a$ is the number of susceptible individuals of age *a*, $I_a$ is the number of infected individuals of age *a*, $R_a$ is the number of removed individuals of age *a*, and $\gamma ^{-1}$ is the infectious period, which is assumed to be the same for all age classes and equal to $\gamma ^{-1}=4.5$ days. COVID-19 is a disease with a more complex natural history than a SIR model can account for, being required to add some pre-symptomatic or asymptomatic compartments, as well as a latency period, for certain applications. Nonetheless, it has been shown that SIR models can correctly describe the overall evolution of the disease [46], which is enough for the scope of this paper. Lastly, $\lambda _a$ is the force of infection for individuals of age *a* and it is expressed as

2 $$\begin{aligned} \lambda _a = \beta \chi _a \sum _{a^{\prime }} M_{aa^{\prime }}\frac{I_{a^{\prime }}}{N_{a^{\prime }}}, \end{aligned}$$

where $\beta$ is the transmissibility of the virus, $N_a$ is the total number of individuals of age *a*, and $M_{aa^{\prime }}$ measures the average number of contacts of an individual of age *a* with individuals of age $a^{\prime }$. Finally, $\chi _a$ is an age-dependent susceptibility factor accounting for the lower susceptibility of children to the disease, i.e. $\chi _a = 0.56$ if $a \le 19$ and 1 otherwise [47].

The basic reproductive number $R_0$ is defined in this model as

3 $$\begin{aligned} R_0 = \frac{\beta }{\gamma }\rho (\chi M), \end{aligned}$$

where $\rho (\chi M)$ is the spectral radius, or largest eigenvalue, of the age-contact matrix (in this case also incorporating the susceptibility factor) [48].

### Scenario

First, we collect the seroprevalence data measured in September 2020 for each age group and US state. We set the corresponding fraction of the population in each state into the removed compartment. Second, we simulate an initial outbreak with an $R_0=1.5$. This basic reproductive number is below the estimated $R_0$ value for unmitigated transmission of the original variant of SARS-CoV-2 that is around 2.5–3 [49]. With this choice, we mimic a scenario in which there are some restrictions, social distancing and other protective measures in place, yielding a smaller effective $R_0$. During this outbreak, we implement a vaccination campaign (described below). By the end of the campaign, all individuals that have not refused vaccine uptake will have been vaccinated.

Once the vaccination campaign is completed, we assume that societies have returned to normalcy, i.e., any kind of restrictions and precautionary measures are lifted. Then, a new outbreak is seeded in each state, emulating a spill over from other states in the country or importation from other countries. In this second outbreak, we set $R_0=6$ which is closer to the dominant variant of concern (delta variant) [50], predominant in the US since mid 2021 until the emergence of the Omicron strain by the end of 2021 [51, 52], being this last variant of concern more highly transmissible with respect to Delta even though with lower pathogenicity [53]. The reason to propose these secondary outbreaks in each US state with an $R_0$ more in line with Delta is the availability of data to compare our hypothetical scenario with reality. Note that we assume that no awareness or other non-pharmaceutical interventions are in place during this outbreak. Thus, it can be thought as the worst case scenario of resurgence after a vaccination campaign.

**Fig. 1:**
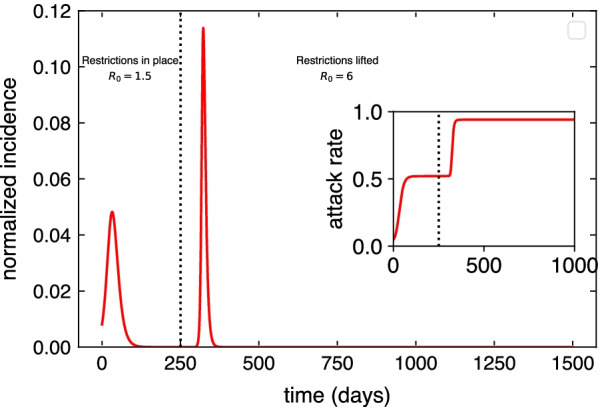
Proposed baseline scenario. Following the first wave of the epidemic, part of the population acquires natural immunity. Then, we simulate the propagation of a mitigated outbreak due to the presence of some restrictions, social distancing and prophylaxis measures, leading to a slower propagation of the original variant of the disease ($R_0=1.5$). After the outbreak extinguishes a back-to-normal situation is assumed and all prevention measures are lifted. Then, an outbreak is seeded again with a higher basic reproductive number, $R_0=6$. On top of this baseline scenario, we will introduce a vaccination campaign during the first outbreak and explore the impact of vaccination hesitancy on the second outbreak

As a visual example of the proposed scenario, in Fig. 1 we show how the incidence would evolve at the level of state for the full epidemic if no vaccination campaigns were deployed during the first outbreak. When an aggressive variant sets in, secondary outbreaks may still cause havoc. The inset depicts the evolution of the prevalence, which can reach almost the whole population for large enough $R_0$. In Sect. 3, we explore how the vaccination efforts modify this baseline scenario.

### Vaccination

We use data from The COVID States Project (https://covidstates.org), in particular the surveys in Report $\#$43: COVID-19 vaccine rates and attitudes among Americans [39]. These surveys provide information on vaccination acceptance/hesitancy by age at the state level. Therein, several degrees of predisposition toward vaccines are reported. The following categories are distinguished: individuals who are “already vaccinated”, individuals who are inclined to be vaccinated “as soon as possible”, “after at least some people I know”, “after most people I know”, and finally people who “would not get the COVID-19 vaccine”. The shares of people in each category is given at a national level for different age groups. The data shows an important amount of heterogeneity in each of those categories by age group. However, at the level of state, the data is not disaggregated by age groups, only the share of people in each vaccine acceptance category is shown. More specifically, we are looking for the coefficients $g_{c, a}^{\text {state}}$, which represent the share of people for every acceptance category, *c*, and age-class, *a* in every US state. These coefficients satisfy $N_c^{state}=\sum _a g_{c,a}^{state} N_a^{\text {state}}$, where $N_a^{\text {state}}$ is the population of the state in the age class *a* and $N_c^{\text {state}}$ is the population of the state in the acceptance category *c*. These $N_c^{\text {state}}$ values are provided in appendix A within the report, but the information is not disaggregated by age at the level of state [39].

The report offers information at a national level about how people are distributed within acceptance categories by age groups. We refer to the shares shown in the report as $h_{c,a}^{\text {national}}$, which are normalized by age-class, that is, $1=\sum _a h_{c,a}^{\text {national}}$ for a particular category *c*. The quantity $\sum _{a} h_{c,a}^{\text {national}} N_a^{state}$ would be the number of people if national coefficients apply for a certain state and vaccine acceptance category *c*. We relate these coefficients $h_{c,a}^{national}$ to coefficients $g_{c,a}^{\text {state}}$ through a linear transformation:

4 $$\begin{aligned} g_{c,a}^{\text {state}}=h_{c,a}^{\text {national}} \frac{N_c^{\text {state}}}{\sum _{a}h_{c,a}^{\text {national}} N_a^{\text {state}}}\end{aligned}$$

This transformation preserves the shares of people in a certain vaccine acceptance category c in every state and also allows for the introduction of age heterogeneity adapted from the national-level data.

Vaccination campaigns are complex and depend highly on several properties of the population: age, risk groups, professions, supplies, infrastructure, etc. Since we are mostly interested in the aftermath after the vaccination campaign, we adopt a simple scheme. From the aforementioned surveys, we extract the fraction of the population within each age group and state that is willing to be vaccinated, $V_a^{\text {state}}$. We set the length of the vaccination campaign to be $\Delta t_v = 150$ days and assume that the fraction of population vaccinated per unit time is constant and equals to $V_a^{\text {state}}/\Delta t_v$. Both susceptible and recovered individuals can be vaccinated. For simplicity, the vaccine is assumed to be 100% effective in preventing the infection.

## Results

**Fig. 2:**
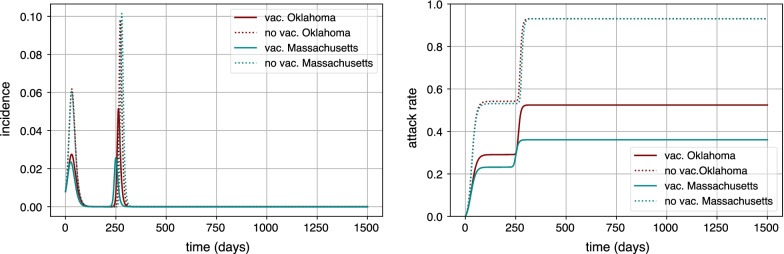
Comparison of spreading dynamics. Comparison of peak incidences and final epidemic sizes for the states of Oklahoma (OK), which has the highest vaccine hesitancy, and Massachusetts (MA), where the vaccine hesitancy is the lowest according to surveys [39]. Continuous trajectories (blue and red) represent the simulation with vaccination campaign, whereas dotted trajectories represent the simulation without introducing the vaccination campaign. All simulations started with a fully susceptible population

In Fig. 2, we show how the incidence and prevalence of the disease changes from the baseline scenario depicted in Fig. 1 when vaccination is in place. In particular, we consider the state with the highest vaccine hesitancy, Oklahoma (OK), and the state with the lowest one, Massachusetts (MA). Additionally, for a fairer comparison, the simulations were started with a null initial condition for prevalence ($R_a(t=0)=0$) (i.e. considering that the whole population is in the susceptible state). Dotted lines in the figure show the case without vaccination. We can see that the impact, in each isolated outbreak and for the full epidemic, is more or less the same for both states, differences owing to population internal structure. When vaccination is introduced in the model (continuous lines), we can appreciate the reduction of peak incidence and epidemic final sizes for both states during the first outbreak. However, when we simulate the second outbreak, the state with the lowest vaccine hesitancy shows a remarkably lower impact, while the other state experiences a sizable second outbreak. The peak of the outbreaks is similar in both outbreaks for Massachussetts, while in Oklahoma, the secondary outbreak is around twice as large as the first outbreak.

Next, we focus on the overall effect of vaccination on the spreading. We explore the relationship between the final attack rate (total fraction of the population that was infected) and the fraction of non-vaccinated individuals in each state. At the time of the surveys vaccine uptake on underage people was not being considered and there was no data regarding the attitudes of this age group. Thus, this set of individuals is composed by both underage people and adults who manifested vaccine hesitancy in the aforementioned surveys [39].

**Fig. 3:**
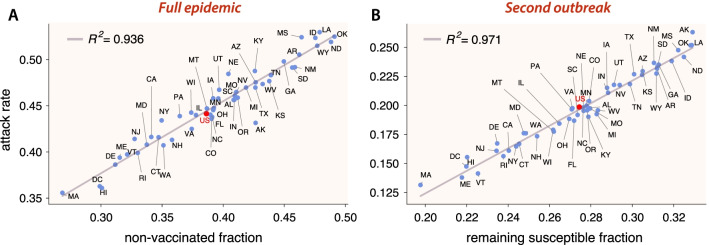
Attack rates scatter plots. Scatter plot of attack rates after the full epidemic (first outbreak with $R_0=1.5$ and the second one with $R_0=6$) versus the non-vaccinated fraction of individuals (**A**), and attack rates of the second outbreak ($R_0=6$) versus the remaining susceptible fraction after the first outbreak (**B**) for every US state. The red dot corresponds to a simulation on a population representing the whole country. It is clearly seen that higher hesitancy translates into higher attack rates

**Fig. 4:**
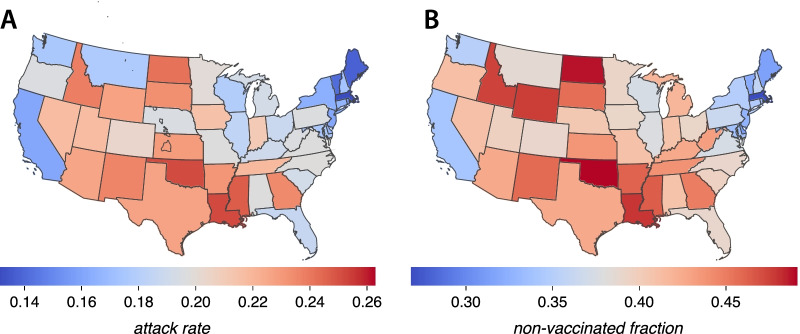
US map. Representation on the US map of the attack rates of every state after the end of the epidemic trajectory proposed in this paper (**A**), and the fraction of non-vaccinated individuals (**B**). Some spatial clustering can be appreciated along the country, even though in the simulations all states are completely isolated

In order to look for a possible correlation between state-level attack rates and the fraction of non-vaccinated individuals, we performed a linear regression. Figure 3A depicts a scatter plot of the attack rates versus the fraction of non-vaccinated individuals for the simulated full epidemic unfolding in every state. The coefficient of determination, $R^2=0.936$, shows a clear relationship between attack rates and vaccine hesitancy for the full period. Note that we have added a simulation for the whole country (the red dot in the scatter plots) with the age-structure from the whole population. Figure 3B shows a scatter plot of the attack rate of the second outbreak, versus the fraction of remaining susceptible individuals at the end of the first outbreak. Here, the coefficient of determination is also very high, $R^2=0.971$. Note that the use of the remaining susceptible fraction rather than directly the fraction of non-vaccinated individuals owes to the fact that once the first outbreak and the vaccination campaign have ended, the demographic structure of the pool of susceptible individuals has changed dramatically. This pool is all conformed by individuals that either declined vaccination or are underage. Since, according to data, hesitancy rates are low in older people, there is a predominance now of younger susceptible individuals. Additionally, Fig. 4 represents the very same data of Fig. 3A on the map of the United States. We observe some geographical clustering, even though we are treating each state as a completely isolated population. The states with higher attack rates or, similarly, the states with a higher fraction of vaccine hesitancy, are concentrated mainly in the interior of the country (inner Pacific west, Intermountain), ranging from north (Midwest) to south (inner Southeast).

Let us next try to get a deeper understanding of what is happening during our simulated second outbreak. Looking at some particular extreme examples, we can appreciate that the state of Massachusetts (MA), with the lowest vaccine hesitancy (9$\%$ of adult population), has the lowest epidemic size during the first outbreak and also during the second outbreak. On the other hand, Alaska (AK) shows one of the lowest attack rates in the first outbreak, but the highest one in the second outbreak, together with the highest fraction of remaining susceptible at the end of the first outbreak, whereas its hesitancy amounts to $23\%$ of the adult population, way behind the most reluctant states. Interestingly, there are other states with a relative low hesitancy rate that also show a sizable second outbreak. This is the case of the state of Utah (UT), with a hesitancy of about $15\%$ among the adult population but nevertheless ranking high in the size of the second outbreak. One could hypothesize that these two states should have a similar number of deaths during the second outbreak. But, remarkably, as we show below, there is indeed more than a simple extrapolation of the correlation between the outbreak size and the number of non-vaccinated/susceptible individuals when it comes to forecasting mortality. The reason is that the age of non-vaccinated and/or remaining susceptible matters, not only because it usually determines behavior (and risk of infection) but also because the infection fatality rate heavily depends on it. As [40] emphasizes, considering transmission through the lens of (age-based) contact patterns is fundamental to understanding which population groups are driving disease transmission. Several reports, at least for the US, point to the fact that transmission dynamics shifted from older adults in the first stages of the pandemic to younger groups later [40, 54, 55]. This is understandable since once the harshest lockdowns were lifted, naturally younger groups are more socially active. In contrast, elders are less active and due to epidemic awareness, one should expect that they mix more carefully. Regarding mortality, it has been well documented the increasing risk of suffering severe disease and death for the oldest age groups, specially beyond 65 years old [41, 56–60].

**Fig. 5:**
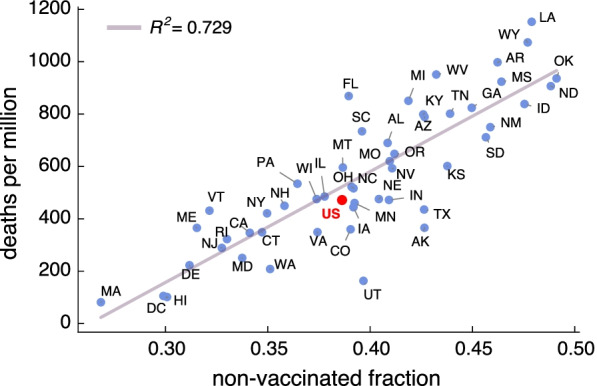
Death scatter plots. Scatter plot of deaths per million during the second outbreak versus the non-vaccinated fraction at the end of the first outbreak for every US state. Results also shown for a simulation of the epidemic for the whole country as if it were a single age-structured population (red dot). The model does not include deaths as part of the dynamics, but they can be estimated by applying the infection fatality rate to the final fraction of individuals in the removed compartment for each age class (Eq. (5))

In Fig. 5, we show a scatter plot of deaths per million individuals in the second outbreak versus the fraction of non-vaccinated individuals at the end of the first outbreak. We estimate the number of deaths in each age group by applying the corresponding infection fatality rate (IFR) [56], so that:

5 $$\begin{aligned} D_a(\infty ) = \text {IFR}_a\times R_a(\infty ), \end{aligned}$$

where $R_a(\infty )$ and $D_a(\infty )$ are, respectively, the prevalence and the number of deceased individuals at the end of a particular outbreak.

Even though a high coefficient of determination is obtained, its explanatory power is smaller than for the attack rate, which suggests that there are other factors playing a relevant role. Certainly, we can appreciate that higher proportions of deaths tend to occur in those states with higher hesitancy. Bringing back the case of Alaska (AK), and contrary to what could be naively expected, we see that it has been overtaken by several states. Even more striking is the case of Utah (UT), being in the lower part of the ranking. This clearly reveals that apart from vaccine hesitancy, the age structure is playing a key role in the disease dynamics and COVID-related fatalities [56, 58, 59].

**Fig. 6:**
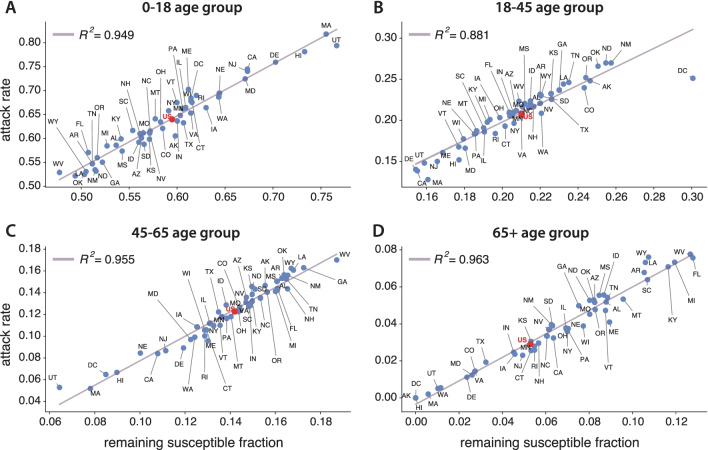
Attack rate scatter plots by age. Scatter plot of attack rates during the second outbreak versus the remaining susceptible fraction for every US state. Top-left (**A**): 0–18 years old group. Top-right (**B**): 18–45 years old group. Bottom-left (**C**): 45–65 years old group. Bottom-right (D): over 65 years old group. Results also shown for a simulation of the epidemic for the whole country as if it were a single age-structured population (red dot). These high correlations show also the relevant role of age structure in the disease propagation

To understand better these interdependencies, we next look at the attack rates during the second outbreak by coarse-graining the 85 age groups resolved in our model into four main relevant categories for the sake of the analysis. In Fig. 6, we show results for 0–18 (A), 18–45 (B), 45–65 (C), and more than 65 year old age groups (D). For each one, the attack rates during the second outbreak are computed as $R_a(\infty )/R(\infty )$, while the fractions of remaining susceptible individuals at the end of the first outbreak are computed as $S_{1a}/S_1$, where $R(\infty )$ is the final attack rate, and $S_1$ is the total fraction of remaining susceptible subjects. Thus, these figures tell us the share of people in each group *a* within the susceptible and removed pools.

## Discussion

The results by age groups exhibit a very high correlation for the linear fittings, which indicate the relevance of age structure in the transmission of the disease. For every age group, states with higher hesitancy tend to experience larger epidemic sizes. Regarding the cases mentioned before, namely, Utah (UT) and Alaska (AK), one can see that their fractions of remaining susceptible individuals are large in the youngest age groups and rather small (null for Alaska) in the 65 + age strata. This ultimately explains why these two states undergo large secondary outbreaks that are not translated into a higher number of deaths. Finally, we also note that the fraction of remaining susceptible individuals is the highest for roughly every state in the two youngest age brackets (around or higher than 50%), which means that the younger age groups will be the driving group of the second outbreak.

It is thus clear the relevance of both vaccine hesitancy and of age heterogeneity in order to project the impact of the epidemic spreading on a territory. To showcase this, we look for an estimation of how many deaths could potentially be averted just by reducing the fraction of individuals in the “would not get the COVID-19 vaccine” category in one percentage point. It may occur that for states with an important share of younger population and not very high hesitancy, an extra effort does not pay off. Conversely, in states with an older population and for those with high hesitancy, such additional increase in the percentage of vaccinated may represent an important benefit. We believe these are important considerations for public health policy making. Table 1 shows the number of averted deaths per million people if vaccine hesitancy is reduced by one per cent during the vaccination campaign in every state. A first look at the table would take us to believe that, overall, states with higher hesitancy will tend to avert more deaths by improving their vaccine roll out. But if we look for correlations between the total number of averted deaths in both outbreaks and the fraction of non vaccinated individuals, we obtain a not so high Pearson coefficient of $\rho =0.61$, signalling correlations but not quite strongly. We have learnt throughout the discussion and related literature review the severe impact that disease has on the eldest groups within a population and specially in this work, the importance of having a low pool of remaining susceptible individuals in the oldest age groups (65 +) for having lower deaths rates in secondary outbreaks. If then we correlate this quantity with the total number of averted deaths, we obtain a Pearson coefficient of $\rho =0.92$ and thus very high correlation and greater explanatory power.

**Table 1:** Average number of averted deaths per million (95% CI in brackets), separately in the 1st and 2nd outbreak, due to reducing vaccine hesitation in one percent point

State	Averted deaths (1st outbreak)	Averted deaths (2nd outbreak)	State	Averted deaths (1st outbreak)	Averted deaths (2nd outbreak)
AK	5.12 [2.83–10.57]	16.78 [9.37–35.14]	MT	12.03 [6.62–23.86]	34.49 [18.93–67.57]
AL	8.62 [4.73–17.13]	39.18 [21.49–76.94]	NC	8.08 [4.44–16.11]	34.3 [18.83–67.7]
AR	10.23 [5.61–20.29]	42.38 [23.17–82.64]	ND	10.83 [5.92–21.52]	35.56 [19.34–69.23]
AZ	9.66 [5.29–19.12]	39.74 [21.73–77.58]	NE	9.75 [5.34–19.35]	27.7 [15.11–54.23]
CA	8.79 [4.81–17.45]	24.96 [13.62–49.08]	NH	12.1 [6.67–24.09]	33.04 [18.19–65.35]
CO	7.66 [4.21–15.43]	26.57 [14.62–53.06]	NJ	4.49 [2.47–8.91]	27.55 [15.08–54.42]
CT	10.43 [5.72–20.71]	28.18 [15.43- 55.57]	NM	10.73 [5.9–21.38]	37.28 [20.45–73.19]
DC	4.09 [2.22–8.28]	9.43 [5.16–20.01]	NV	7.57 [4.16–15.1]	34.65 [19.04–68.39]
DE	8.34 [4.59–16.6]	26.71 [14.71–52.98]	NY	4.18 [2.29–8.29]	31.78 [17.35–62.54]
FL	10.3 [5.62–20.2]	48.74 [26.54–94.48]	OH	10.55 [5.79–20.98]	32.53 [17.83–63.96]
GA	5.89 [3.24–11.8]	38.9 [21.38–76.83]	OK	9.74 [5.34–19.41]	38.24 [20.93–74.83]
HI	9.31 [5.09–18.44]	10.87 [6.06–22.76]	OR	11.05 [6.07–21.97]	35.75 [19.61–70.23]
IA	7.31 [4.0–14.56]	31.54 [17.25–62.05]	PA	7.84 [4.3–15.2]	36.33 [19.88–71.2]
ID	10.18 [5.59–20.29]	35.19 [19.29–68.9]	RI	10.26 [5.63–20.38]	26.66 [14.59–52.55]
IL	9.28 [5.08–18.44]	30.61 [16.73–60.12]	SC	9.66 [5.32–19.19]	39.87 [21.91–78.17]
IN	9.00 [4.95–18.04]	30.06 [16.51–59.43]	SD	10.53 [5.79–21.03]	35.13 [19.24–68.87]
KS	9.08 [4.99–18.16]	33.38 [18.29–65.69]	TN	9.54 [5.24–19.0]	39.14 [21.47–76.84]
KY	11.88 [6.52–23.52]	37.12 [20.32–72.48]	TX	5.99 [3.29–12.05]	27.89 [15.3–55.49]
LA	7.68 [4.22–15.25]	45.27 [24.79–88.51]	UT	5.68 [7.38–26.53]	17.02 [9.31–34.05]
MA	8.37 [4.59–16.67]	16.94 [9.29–33.83]	VA	8.31 [4.57–16.66]	27.93 [15.37–55.55]
MD	5.64 [3.1–11.27]	25.95 [14.27–51.71]	VT	13.40 [7.38–26.53]	31.36 [17.21–61.56]
ME	14.13 [7.77–27.89]	29.7 [16.29–58.21]	WA	8.14 [4.48–16.35]	22.54 [12.4–45.11]
MI	12.03 [6.6–23.82]	40.84 [22.34–79.66]	WI	10.70 [5.88–21.28]	30.5 [16.71–59.9]
MN	7.42 [4.08–14.82]	31.34 [17.18–61.9]	WV	13.25 [7.28–26.2]	44.72 [24.5–87.12]
MO	10.60 [5.81–21.04]	34.9 [19.09–68.37]	WY	12.39 [6.82–24.64]	39.78 [21.81–77.73]
MS	8.78 [4.82–17.48]	38.48 [21.09–75.36]	US	7.95 [4.36–15.85]	32.15 [17.62–63.43]

Results shown for every state and for the whole country (US)

The results shown in this work are based on a standard and sound epidemiological approach based on compartmental ODE modeling with a heavily-based data-driven input for several aspects: population age structure, mixing patterns by age, and vaccine uptake attitudes. The proposed scenario on which the model is run, however, does not maps exactly any real situation experienced in the US and thus our aim was not to reproduce neither forecast accurately realistic COVID-19 trajectories. The particular outcomes brought should be regarded as what-if scenarios or hypothetical outcomes of what to expect overall given the premises hold. If, for instance, vaccine acceptation fractions should differ, as well as the transmissibility of a given virus strain, the specific figures could change dramatically, but not so the underlying conclusions. Additionally, from the beginning the model confection and the devised scenario was not conceived to realistically simulate or reproduce the myriad of complexities and heterogeneities involved in COVID-19 spreading country-wise. However, we can still check to a certain point how the model performed with respect to reality and thus gain confidence on the conclusions derived from it or rather discard the approach as insufficient or unsatisfactory.

As explained above, we proposed a hypothetical situation in which the epidemic spreading was ongoing but under rather mild transmission conditions due to disease awareness and general restrictions, while at the same time a mass vaccination campaign was deployed. After vaccination was completed and the epidemic wave was rather halted, then we simulated that societies turned back to normal from very low daily incidence, but the virus was still there and new secondary outbreaks emerged.

**Fig. 7:**
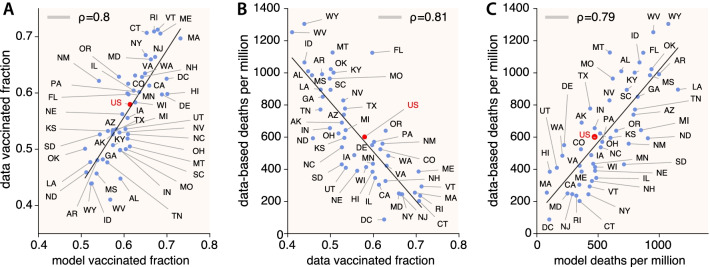
Comparison of model output and real data for the Delta wave in the US. Correlation analysis (Pearson correlation coefficient) for data and model observables. Left **A**: Data vaccinated fraction until 31/10/2021 versus model/survey vaccinated fraction. Center **B**: Data based deaths per million versus real vaccinated fraction. Right **C**: Data based deaths vs. model deaths per million. High correlations are obtained between the model output and real data

In reality, vaccine roll out in the US took off in the beginning of 2021 among concerns about vaccine hesitancy, a few months before we posed our research questions and designed the aforementioned hypothetical scenario. By mid February the highest epidemic wave experienced by the country until that moment was ending and reaching a plateau of rather low incidence. Overall, the situation stayed under control except for a very slight peak around mid April and then a relaxed decrease until reaching the lowest national incidence levels by June 2021 since the beginning of the pandemic. Thus, we could draw some parallelisms here with our first wave in our experiment: a rather controlled and decaying progression with vaccination going on. Then, restrictions were overall lifted up and a new wave started to build up by the beginning of July 2021. This wave was mainly driven by the Delta variant of concern, peaking on the first days of September, reaching a higher plateau than the previous wave by early November 2021, and immediately followed by the huge Omicron wave. Given the history of the spreading dynamics in the US, we consider that our hypothesized secondary outbreaks in every state, describing a rather unmitigated scenario with a more aggressive strain, could match reasonably well the Delta wave that took place across the described period. Therefore, it is informative to check how the model output relates to the real impact of the epidemic during the aforementioned period. In Fig. 7, we looked at the following pairs of observables to check for correlations: real data [61] vs. model/survey vaccination fractions (Fig. 7A), data-based deaths [62] vs. real vaccination fractions (Fig. 7B), and data-based deaths vs. model deaths (Fig. 7C). We can see that all confronted observables show a high correlation. First, in panel A, comparing data and model vaccination fractions we obtain a Pearson correlation coefficient of $\rho =0.8$. Overall, we can say that surveys on attitude towards vaccination were good, strengthening subsequent model results. Second, in panel B we obtain a value of $\rho =0.81$ when correlating what happened in reality regarding deaths and vaccination. As expected, the higher the vaccination fraction in a state, the lower the deaths that took place due to the COVID-19. When comparing in the paper the model deaths per million with the non-vaccinated fraction (for the matter, this is equivalent to comparing it to the vaccinated fraction), we obtained an $R^2=0.729$ and therefore a Pearson coefficient of $\rho =0.85$, a very high signal of correlation. We can then see that our model projections captured the real trend quite satisfactorily and this emphasizes the dominant role of the vaccine of mitigating the impact on the population. Finally, in panel C we obtained a Pearson coefficient $\rho =0.79$ when comparing this time the real data-based estimation of deaths and the model-based estimation of deaths. Thus we find again a high correlation between our hypothesized scenario of secondary outbreaks and the Delta wave.

## Conclusions

In this work, we have explored SARS-CoV-2 transmission dynamics on a population that is partially vaccinated and is seeded again with the virus when restrictions are fully lifted. We explored, in particular, to what extent vaccine hesitancy may still drive sizable outbreaks in a context where a more transmissible SARS-CoV-2 variant of concern is dominant. We used data from vaccination acceptance surveys, together with up-to-date age distributed populations and contact matrices in the US to inform an age-structured SIR model.

Our results show a clear correlation between the size of experienced outbreaks, once all kinds of measures are lifted, and the fraction of vaccine hesitancy or, similarly, the fraction of remaining susceptible individuals at the onset of a second outbreak. Higher vaccine hesitancy ratios expose the population to larger outbreaks and, inversely, higher vaccine acceptance ratios can mitigate the impact to the point of negligible secondary waves due to immunity of the population.

We have also inspected in detail the role of the age structure of the population in both the attack rate and the mortality of secondary outbreaks. Our findings reveal that the prevalence is highly correlated with the fraction of remaining susceptible individuals by age classes, with the youngest contributing the most to the attack rate. It is however not immediate to project such a correlation to the expected number of deaths, as here too age plays a role, though in the opposite direction, e.g., the younger the population, the lower the mortality.

Lastly, we estimated the number of potentially averted deaths during the course of the simulated epidemic if the number of people reluctant to vaccine uptake were reduced in one percentage point. Results show again the relevance of regarding age structure in transmission since not all the states with higher hesitancy rank highest in averting deaths. It is very relevant the fraction of hesitant individuals in the older groups.

To round up the analysis, we investigated how the model fared when comparing with real data. Even though the devised experiment here was not intended to accurately replicate or forecast real COVID-19 trajectories, the data-driven approach and sound modeling offered very high correlations when comparing survey/model vaccination against real vaccination, and model death estimation against real data-based deaths during the Delta wave in the US.

We acknowledge that our model has several limitations. One is at the core of its compartmental structure, not including a more detailed progression of the natural history of the disease, which might affect our estimation of deaths, and does not consider hospitalizations of any kind. The vaccination campaign could be implemented in a more realistic way and owing to each state idiosyncrasy but, more importantly, vaccines are revealing to be not sterilizing and thus not fully preventing transmission and, on top of that, immunity decays over time. These facts do not affect the overall dynamics explored in this paper, but should be incorporated to provide reliable estimations on the exact amount of expected infections or deaths. Additionally, the behavioral responses are not completely accounted for. All these factors open important challenges for future works.

To conclude, the most important implications of the results reported here include: (i) data on vaccination by age is important to accurately capture the evolution of mortality in secondary waves; (ii) surveys on vaccination attitude are a valuable proxy to estimate the hesitancy of the population; (iii) allocation of additional resources is more important in states with relative high hesitancy rates but specially in states where the remaining susceptible population is older; (iv) reintroduction of restrictions could be needed in states with very high attack rates to reduce pressure over healthcare systems; and (v) incentives to vaccination will reduce the number of deaths if they focus on the older generations.

## Data Availability

All the data used in this paper is publicly available. The age-contact matrices can be directly downloaded from https://zenodo.org/record/4287574 and the information on vaccination attitudes is contained in the tables of the Appendix A of https://osf.io/rnw8z/.
